# Inside the ‘imperfect mosaic’: Minority ethnic women’s qualitative experiences of race and ethnicity during pregnancy, childbirth, and maternity care in the United Kingdom

**DOI:** 10.1186/s12889-023-17505-7

**Published:** 2023-12-21

**Authors:** Sergio A. Silverio, Nila Varman, Zenab Barry, Nina Khazaezadeh, Daghni Rajasingam, Laura A. Magee, Jacqueline Matthew

**Affiliations:** 1 Department of Women & Children’s Health, School of Life Course & Population Sciences, Faculty of Life Sciences & Medicine, King’s College London, Addison House, Great Maze Pond, Southwark, London, SE1 1UL UK; 2 National Maternity Voices, London, UK; 3 National Institute of Health and Care Research Applied Research Collaboration [NIHR ARC] South London, King’s College Hospital NHS Foundation Trust, Lambeth, London, SE5 9RS UK; 4 Chief Midwifery Office, NHS England, Wellington House, 133-155 Waterloo Road, Southwark, London, SE1 8UG UK; 5 Maternity Services, St. Thomas’ Hospital, Guy’s and St. Thomas’ NHS Foundation Trust, Westminster Bridge Road, Lambeth, London, SE1 7EH UK; 6 Department of Perinatal Imaging & Health, School of Biomedical Engineering & Imaging Sciences, Faculty of Life Sciences & Medicine, King’s College London, St. Thomas’ Hospital, Westminster Bridge Road, Lambeth, London, SE1 7EH UK

**Keywords:** Race, Ethnicity, Equity, Discrimination, Maternity care, Obstetrics, Midwifery, Qualitative research, Interviews, Grounded theory, The NHS, Women, Women’s Health, Empowerment, Health Services Research

## Abstract

**Background:**

Persistent, high rates of maternal mortality amongst ethnic minorities is one of the UK’s starkest examples of racial disparity. With greater risks of adverse outcomes during maternity care, ethnic minority women are subjected to embedded, structural and systemic discrimination throughout the healthcare service.

**Methods:**

Fourteen semi-structured interviews were undertaken with minority ethnic women who had recent experience of UK maternity care. Data pertaining to ethnicity and race were subject to iterative, inductive coding, and constant comparison through Grounded Theory Analysis to test a previously established theory: *The ‘Imperfect Mosaic’.*

**Analysis & findings:**

A related theory emerged, comprising four themes: ‘Stopping Short of Agentic Birth’; ‘Silenced and Stigmatised through Tick-Box Care’; ‘Anticipating Discrimination and the Need for Advocacy’; and ‘Navigating Cultural Differences’. The new theory: *Inside the ‘Imperfect Mosaic’*, demonstrates experiences of those who received maternity care which directly mirrors experiences of those who provide care, as seen in the previous theory we set-out to test. However, the current theory is based on more traditional and familiar notions of racial discrimination, rather than the nuanced, subtleties of socio-demographic-based micro-aggressions experienced by healthcare professionals.

**Conclusions:**

Our findings suggest the need for the following actions: Prioritisation of bodily autonomy and agency in perinatal physical and mental healthcare; expand awareness of social and cultural issues (i.e., moral injury; cultural safety) within the NHS; and undertake diversity training and support, and follow-up of translation of the training into practice, across (maternal) health services.

## Background

Issues of race and ethnicity continue to play a role in the debate on maternal health disparities and perinatal inequity in the United Kingdom. Recent evidence from maternal and child healthcare professionals suggests the nature of racial issues, systemic racism, and structural ethnic discrimination is taking different and less obvious forms, but remains extant [1]. Minority ethnic women accessing maternity care through the NHS, are more likely to experience adverse outcomes than their White counterparts [2–6], with maternal mortality being four-times higher amongst Black women, almost twice as high in Asian women, and just under 1.5 times as high in Mixed Ethnicity women, than White women [2]. The persistent, high rates of maternal mortality amongst minority ethnic women is not only stark, but represents one of the most damning indictments of maternal healthcare in the UK. Outcome disparities are not due simply to biological or social health factors inherited by ethnic minority women, but also to poor quality and unsafe care, and sometimes, mismanagement [2, 3]. These disparities were magnified during the pandemic, as the SARS-CoV-2 virus disproportionately affected those in Black, Asian, and Minority Ethnic groups [7], who were also less likely have been vaccinated against COVID-19 [8–10].

Injustices derived from racial and ethnic disparities have long been prevalent in the NHS, and continue to attract significant commentary. Despite a growing evidence base and renewed efforts to affect positive policy and practice change, ethnic minority (vs. White British) women continue to report poorer maternity care experiences and birth outcomes, and greater mistrust in UK health services [4–6]. Furthermore, updated policy and practice guidance frequently exacerbates inequalities [3, 11]. While there are official reports stating structural and systemic racism is rare or absent within the NHS [12], contemporary empirical and practice publications and commentaries continue to refute this and highlight that recent health system shocks (such as those caused by Brexit, the SARS-CoV-2 pandemic, and the NHS-wide staffing crisis) have exacerbated discrimination against ethnic minorities within the NHS [13–19], especially for those involved in providing or receiving maternity care [1, 11].

The changing nature of prejudices extant within maternal and child healthcare services is a relatively new addition to the field of debate. Our recent analysis of healthcare staff who *provided* care during the pandemic found ongoing prejudices are more nuanced than traditional and familiar notions of racism based on skin colour, and extend to other socio-demographic factors [1]. Micro-aggressions (i.e., behaviours which are not an exertion of direct aggression towards another, but are snide, underhand, or disruptive towards another) between so-called ‘in-groups’ and ‘out-groups’ bring into sharp focus the notion of identity, and as seen in recent work with healthcare professionals in the UK, the concept of ‘Britishness’ [1]. In that analysis, the NHS was compared to an ‘Imperfect Mosaic’ – inflexible, lacking plasticity, and generally maladapted to overcome issues centred on race and ethnicity amongst its staff, choosing instead to ‘paper over the cracks’ in an increasingly-pressured system of service delivery, made worse by the global pandemic [1].

Though rarely conducted, ‘testing’ of a previously established Grounded Theory can assist our understanding about whether experiences of specific phenomena (such as experiences of race and ethnicity), across specific contexts (such as maternity care), are shared by different populations [20, 21]. We aimed to test the previously established theory (The ‘Imperfect Mosaic’) and, as such, we report an analysis of interviews with women who *received* maternity care in the UK, to test how their experiences compare with the previously-published theory, the ‘Imperfect Mosaic’, which was developed with interview data from maternity healthcare professionals who *provided* maternity care during the pandemic in the UK [1].

## Methods

### Details of ethical approval

Ethical approvals for The Rep All Women Study were granted by the King’s College London Research Ethics Committee (reference HR/DP-20/21-21756; June 2021). Prior to their interviews, all respondents consented to be recorded and for their data to be used in subsequent academic work (e.g. reports; theses; publications; conference dissemination).

### Study design

This qualitative analysis forms part of a nested interview study within the wider ‘Representation in UK Pregnancy Scanning Research: Priorities, Perceptions, and Experiences of All Women’ Study (The Rep All Women Study). Specifically, interview data about minority ethnic women’s experiences of race and ethnicity whilst utilising maternity care, formed the basis of the present analysis. Data collection and analysis followed best-practice procedures for qualitative research into sensitive, challenging, and difficult topics [22].

A post-positivist research paradigm was adopted [23], underpinned by the philosophy of a critical realist ontology (whereby acquired knowledge may in fact be fallible), and an objectivist epistemology (whereby even knowledge which is falsely recalled allows for an understanding of the lived reality) [24]. A lifecourse analysis approach is helpful when adopting this type of philosophy, as it takes account of extant social contexts which may lens lifecourses which deviate from the norm [25], but is accepting of an individual’s lived reality as a ‘truth’. In terms of positionality, an objective-outsider stance was taken within the data, as none of the researchers had experienced receiving maternity care recently; but we adopted an empathic reflexive judgement about the data; due to some of the researchers being mothers, non-healthcare professionals, and others practicing clinicians.

None of the authors identified as White British. Analyses were conducted in consultation with a peer researcher [ZB] who was trained by the study team to conduct interviews for research, and also shared the lived experience of being a service user from a minority ethnic group. The peer researcher was also a representative of The Rep All Women Community Advisory Group and their involvement in data collection and analysis was used *in lieu* of ‘member-checking’, to ensure interrogation of the interpretation of findings and clarity of presentation.

### Recruitment and participants

Women responded to adverts on social media and on collaborating partners’ websites for The Rep All Women Study on-line survey, and had the option to leave their details to be contacted for an interview, if they met certain inclusion criteria (n = 9). Snowball and word-of-mouth recruitment also took place with interested, prospective participants, e-mailing the study team directly (n = 5). Potential participants were contacted via e-mail by members of the study team for interviews to be arranged. One prospective participant consented to being interviewed, but did not attend the scheduled interview time. All participants identified as non-White British, and whilst some provided a self-identified ethnicity, others preferred to not disclose exact ethnicities. Likewise, participants were free to not disclose any demographic characteristics without providing a reason (see Table 1).

**Table 1:** Demographic characteristics

ID	Self-Reported Race/Ethnicity	Age	Sexual Orientation	Marital Status	Employment Status
1	Asian: British Indian	40	Heterosexual	Married	Maternity Leave
2	Asian: White and Indian	45	Heterosexual	Married	Yes - Full Time
3	Turkish Cypriot	33	Heterosexual	Single	Yes - Part Time
4	Black: British Caribbean	38	Bisexual	Co-habiting	Maternity Leave
5	Black British	39	Not reported	Married	Yes - Part Time
6	Asian British	31	Heterosexual	Married	Unemployed
7	Black	36	Heterosexual	Not reported	Not reported
8	Asian: British Vietnamese	33	Heterosexual	Married	Yes - Full Time
9	Black: British Caribbean	30	Heterosexual	Married	Yes - Full Time
10	Somalian	Not reported	Not reported	Married	Yes
11	Asian: Indian	35	Heterosexual	Married	Maternity Leave
12	Asian: Indian	35	Not reported	Married	Maternity Leave
13	Asian: British Indian	29	Heterosexual	Married	Yes - Full Time
14	Mixed: Caribbean, West Indian, and East Indian	40	Heterosexual	Married	Yes - Part Time

### Data collection

Fourteen interviews were conducted between December 2021 and February 2022, using video-conferencing software [26] to adhere to Government guidance for physical distancing during the pandemic. Interviews were undertaken by the study team which included academic researchers [JM; SAS], an MSc student supervised by the academic researchers [NV], and a peer researcher [ZB].

Use of semi-structured interviews provided a template for common questions to be asked of all participants, whilst allowing enough flexibility for detailed experiential narratives to be recalled [27]. In turn, analysis was focused on the individual ‘lived experiences’, providing rich, layered, and complex understanding of phenomena which would otherwise be difficult to capture quantitatively or with more structured questioning qualitatively [28]. Participants were probed in response to their answers, so we could understand: (i) the psycho-social interplay between them as women receiving maternity care, and the healthcare professionals who provided that care; (ii) the healthcare settings in which they received care; and (iii) women’s experience of the NHS more broadly. As such, our assessments were of micro-level, meso-level, and macro-level interactions. Interviews lasted for 30–55 min (*M*_*Time*_*=*37 min), and were recorded, audio-transcribed, and anonymised.

### Data analysis

As in the work preceding this [1], analysis was centred on participants’ answers to a direct question about the intersection of racial and ethnic backgrounds, maternity care experiences, and participation in maternity-related research asked in every interview and responded to by all. However, all other references to experiences of the intersection of race and ethnicity in maternity care throughout each interview were also identified [NV] and analysed [NV; SAS; JM] – to allow for complete analyses to be undertaken [29].

The process for Grounded Theory Analysis [30], appropriate for cross-disciplinary health research [21], was followed, meaning it was both inductive and iterative, was consultative, and relied on the principle of constant comparison between transcripts as analysis progressed. ‘Line-by-line coding’, followed by ‘focused coding’ (applying codes of more conceptual weight to larger areas of transcript data) was conducted [NV], with input from more senior researchers [SAS; JM] who assisted in turning focused codes into ‘super-categories’ (or emergent, ‘lower-order themes’), before final ‘themes’ were identified – the interaction of which enabled emergence of the final theory: ‘Inside the Imperfect Mosaic’. Throughout the analytic process, scrutiny of codes, super-categories, themes, and the theory was conducted through ‘within-team defence’ (a thorough interrogation of the theory, amongst the different disciplines and PPIE members of the team) [21].

Two principles of data saturation had to be satisfied to cease recruitment and analysis, respectively: (i) data saturation [31], where the same type of data is identified across most or all of the transcripts in the dataset and no exceptionally different concepts were emerging from new interviews undertaken; and (ii) theoretical saturation [32], which is where themes are adequately supported by data to develop a theory. These forms of saturation were achieved at 11 and 14 participants, respectively. The relatively small sample size at which saturation occurred is consistent with data high in specificity [31], indicative of high levels of cohesion between participants’ responses [33], and resulting in sufficient support for themes to emerge with relatively low numbers of interviews [34].

## Analysis & findings

Having tested the theory: An ‘Imperfect Mosaic’ in this new population, a related theory emerged: ‘Inside the Imperfect Mosaic’. The experiences of women from minority ethnic backgrounds echoed those previously presented by healthcare professionals, with regard to race and ethnicity in the NHS [1]; however, women’s experiences were reminiscent of more traditional and familiar notions of discrimination. The discrimination women reported could be broadly categorised into four themes: (1) ‘Stopping Short of Agentic Birth’; (2) ‘Silenced and Stigmatised through Tick-Box Care’; (3) ‘Anticipating Discrimination and the Need for Advocacy’; and (4) ‘Navigating Cultural Differences’. Illustrative quotations are provided for each theme below, with the participants’ self-identified ethnicity (where provided) in parentheses.

### Stopping short of agentic birth

Women expressed the need to recognise and educate themselves about agency and control during pregnancy to make informed decisions. However, more often than not, this was met with uncertainty, doubt, and sometimes tense debate between women and their healthcare providers, leading women to question who had control over their birth experience:

*“It’s your birth. It’s what you want from your body, and I know it’s a particularly important time for me… in the past there were there were times when I wasn’t sure who had the final say about what’s going to happen.”* (P4: Black: British Caribbean).

Women from minority ethnic groups described being subject to so-called urgent procedures, without them having been explained in sufficient detail, and despite their reluctance or protest being disregarded:

*“…they started talking about induction and I said I don’t want one. But the lady booked to anyway… Without the education, I received from her [Doula], I don’t think I would have had the backbone to sort of put my foot down and say: ‘No, I don’t want that. This is still my body, it’s my choice and my daughter is not showing any signs of distress’.”* (P9: Black: British Caribbean).

*“…I was tearful because that wasn’t like what [we] planned. And then I just asked the midwife what she would advise… I sort of went along with their suggestions…”* (P11: Asian: Indian).

Often women in this study sharply contrasted their experiences with other birthing women who they identified as White. Women anticipated disproportionately traumatic experiences, and described feelings of powerlessness, defeat, and loss of all agency to their healthcare providers:

*“She was being wheeled down to have a planned C-section. And she was a white woman, white family… it’s all planned so you know, it’s you know, it’s nothing. It’s obviously still, I guess going to be scary, but it’s not going to be anywhere near as traumatic as [my] experience… mine was unplanned”.* (P3: Turkish Cypriot)

### Silenced and Stigmatised through Tick-Box Care

Many women anticipated racial bias perpetuated by healthcare professionals. Women reported feeling the need to be the ‘perfect’ maternity care patient, especially when they witnessed other ethnic minority women being stigmatised against and silenced:

*“…next to me, was a woman of colour who was expressing herself with her voice, as you do right? Going through the stages of giving birth, and the midwife told her to shut up… she was not making too much noise, and in the next room there was a Caucasian woman making noise and screaming and nope, nobody seemed to ask this woman to.”* (P14: Mixed: Caribbean, West Indian, and East Indian).

This adoption of the ‘perfect’ patient role, was often to ensure they were not neglected, especially if an emergency arose when birthing:

*“There were elements of my general care with my midwives where I had to be overly pleasant and nice and friendly and approachable. So, lots of smiles and lots of kind of positive body language because... I felt like I needed them on my side so when the time came for the birth or for me to need their support. I did feel that pressure as a Black woman to be extra nice to be extra friendly so that they would give me basic care… Basically, when it was time to give birth and not to be seen as aggressive or the angry Black woman… So, if I do show any kind of frustration or anger or for whatever reason, maybe the pain, or maybe the experience, whatever they, they still care for me. I’m assuming that maybe White women did not have the same worries or concerns. They may not have to be overtly friendly and outgoing and affable.”* (P10: Somalian).

Some women commented on the apparent nonchalance with which they were cared for by healthcare professionals, as compared to other women they identified as being of White British heritage:

*“…I called someone to come. They kind of casually came and was like: 'It’s nothing. You know it’s not a big problem. It is fine, just flip him over’. But when she had the exact same problem [choking] with her baby, everybody came running with trolleys and all these machines and so on and I was like: ‘What?’ There was this White British family and then you’ve got me with his father who’s Black, my mixed-race child and me. I’m not White and so I felt like nobody really cares about us, and despite the fact that there’s probably more risks associated with my baby… her baby seemed perfect…”* (P3: Turkish Cypriot).

Not being taken seriously or believed, especially in relation to concerns or pain, was frequently reported:

*“…Black women really must fight to get pain relief or to get pain support because they’re seen as generally more able to tolerate pain than White people, White women, so that also made me question, you know, if I wasn’t from this race. If I wasn’t Black, would I have, would I have to fight as much as I did? My husband had to fight. I had to fight…”* (P10: Somalian).

In some cases, women from this study almost explained away this tick-box approach to care, as being a convenient (un)intentional shortcut for busy, over-extended healthcare professionals to meet their job demands:

*“…for them, if you categorize people in boxes, it makes life a lot simpler to do that right? Instead of getting to know every single person, you’re boxing them into categories. But you need to be aware of your own prejudice. It’s part of maybe, her day-to-day job and she said this so many times that she’s become desensitized to it… that there’s a human being there. but this is someone’s life that they’ve had to go through… so they need to show some empathy and some sympathy for a start”* (P10: Somalian).

### Anticipating Discrimination and the Need for Advocacy

Women in this study described more broadly the other socio-demographic factors (such as immigration, socio-economic, and first language statuses) which minority ethnic communities face when accessing healthcare, which could have a detrimental effect on expressing themselves during care, self-advocating, or drawing on support from external advocates (often for lack of access to them or knowledge about them):

*“I don’t quite feel like they understand what identifying with an ethnic minority is. I’m not sure if there is value in it anymore.”* (P8: Asian: British Vietnamese).

A lack of advocacy within maternity care settings sometimes meant past medical histories and current social complexities were overlooked, leading to potentially dangerous medical and social situations:

*“…I’d be crying, and I just felt like nobody was supporting me and nobody was checking my file properly to know that there was domestic violence involved and so on… I just felt really overlooked. Like my baby’s life didn’t matter as much as that White baby’s life over there… hers was a planned C-section. Mine was a failed C-section. My epidural failed and I felt them cut me open, which is why they then had to stop and see if I was under, and it was really traumatic and so you know, it was really bad, and yet they weren’t supporting me…”* (P3: Turkish Cypriot).

Where advocacy was not or could not be initiated – either by women themselves, or by a trusted, identified birth companion – anticipated discrimination occasionally translated into situations reported as uncomfortable, or worse still, unconsented:

*“When I was going for my early pregnancy scans with my first pregnancy when they thought it was ectopic……… They said we have to do a transvaginal ultrasound. I was so scared, and I was laying there, and I told the person who’s doing it, I said: ‘I’m… I’m really scared’ and then, like: ‘I’ve never experienced anything like this before’. And the person said to me: ‘Well, you’ve had sex to be pregnant, so…’ And I was like: ‘Oh my goodness’; like even things like that, that I think, this is ’cause I think there’s just distrust, yeah.”* (P6: Asian British).

When there was a strong advocate – either brought in by the women themselves, or if one of the healthcare providers themselves stepped into that role, women reported feeling safer, calmer, and experiencing less discrimination:

*“The midwife we got for the drip was incredible. She really listened to us. She was really clear about what... about what we wanted, and she was really respectful, and she backed away and gave us a space we needed which was what I wanted, to be quite active, and so she was great.”* (P4: Black: British Caribbean).

### Navigating Cultural Differences

Women discussed the importance of being able to relate to their healthcare providers. They felt they could place more trust in those from a similar religion or race, or a different but minority ethnic background.

*“Having someone like yourself and having a conversation with you. That’s a human element for me and seeing a woman of colour… I mean that’s relatable and, and… there’s a human element that’s really important, if you’re culturally aware and you don’t just see them as a stereotype. So, you understand that this is a person.”* (P14: Mixed: Caribbean, West Indian, and East Indian).

Also mentioned in parallel was the fact they had less confidence that they would receive authentic care from healthcare professionals who may or may not be from an ethnic minority background:

*“I have experienced – even as a person of colour – that there is a weird kind of racism vibe even in hospital and care settings like this, that I think another one is distrust. I don’t… I don’t know that I would fully trust somebody even as a person of colour. Just to believe what I’m going through, you know? There’s just distrust, So, I think, ’cause women believe there’s a lack of access to support and help and authentic help. So, I think it’s distrust.”* (P6: Asian British).

Often, women reported having attempted to navigate ethnic differences within their maternity care settings:

*“As much as I’m British born and I’m able to communicate with everybody and... there were certain cultural differences that I felt could be getting in the way.”* (P1: Asian: British Indian).

This was sometimes achieved through masking or playing down their racial identity, in order to relate with their healthcare providers in an attempt to eliminate any racial or cultural tension:

*“They could treat me in a way that would be detrimental because I yeah, I just don’t trust that I would be necessarily treated fairly… just make sure you know what it is you’re getting into. Protect yourself, make sure you’re safe. I know that it might sound like a lack of trust or whatever, but I think that that’s what you have to do because otherwise, things can happen and yeah.”* (P5: Black British).

## Discussion

### The theory and framing it within the literature

As is standard, a Grounded Theory is tested by running a similar study with a different population, phenomenon under evaluation, or context. In this analysis, we changed the population (from healthcare providers to mothers), while keeping constant the phenomenon (experiences of race and ethnicity) and context (maternity care). The resultant theory: ‘Inside the Imperfect Mosaic’ elucidates how the patient perspective looks with regards to race and ethnicity within the NHS and – more specifically – within UK maternity services. Whilst our theory echoes that developed with healthcare professionals [1], the findings from women’s experiences are more reminiscent of traditional notions of discrimination, based on skin colour and racial difference, rather than experiences reported by maternity healthcare professionals, regarding more nuanced practices of micro-aggressions and exclusion based on the lack of ‘Britishness’ [1]. This suggests whilst healthcare professionals themselves may be more accepting of racial difference amongst colleagues, they are still – even if unconsciously doing so – foregrounding racial difference amongst the patients they see, as reported by the women in the present study.

It is also important to contextualise our findings amongst other theories and models extant within the literature and given the context of maternity care in the United Kingdom. For example, women who participated in our study noted racialised discrimination and even direct acts of racism along with often reporting perceived sub-optimal care, something which has previously been suggested to contribute to poor outcomes in UK maternity care [35, 36]. Agency in maternity care is an oft-debated facet relating to quality and safety, as is the concept of relational care whereby women report being both genuinely listened to and taken seriously upon complaint or query. These factors were highlighted in our study with minority ethnic women reporting a loss of agency during birth or comparatively less agentic birth experiences than other non-minority ethnic women they witnessed or knew; as well as experiencing the notion of being silenced. Previous research in the UK [37] has reported British-born minority ethnic women to be more in control of their care (something not reported by women in our study), but still stereotyped according to their cultural heritage (which appeared as very familiar discourse in our work). Furthermore, cultural dissonance has been reported as an explanation for the institutional, interpersonal, and internalised racism which Black, Asian, and Minority Ethnic women face [38], again speaking to the tick-box and stigmatised maternity experiences as reported by women in our study. Other work with migrant women [39] has demonstrated the importance of authoritative knowledge and communication of information between midwife and woman traversing family relationships, the healthcare system itself, and embedded religio-cultural factors; which speaks to our themes highlighting the need for advocacy and navigating cultural differences.

### Summary of main findings

Potential implicit bias held by maternal healthcare staff may work against the building of rapport between clinicians providing care and the women who seek it, as well as distorting communication and removing women’s agency over care decisions. Our data suggest that having reassurance that they do indeed have control over the healthcare decisions made, might encourage more trusting relationships between women and their healthcare providers, and encourage women to express their care needs. We emphasise, as others have before us [40], that encouraging and respecting women’s agency during pregnancy and childbirth is a pivotal part of providing effective, unbiased, and informed care, which is both safe and woman-centred.

Erroneous stereotypes, implicit biases, and negative perceptions may influence the way minority ethnic groups are regarded by healthcare professionals. Some of the women who participated in the study felt as though they were being perceived and treated as sub-human, which led them to question their own worthiness for holistic care – something which has been found in previous research [41, 42]. Moreover, the reporting by women of their experiences of being silenced and dismissed, echoes previous research [43], and concerns raised about the NHS being impermeable to critique about its flaws [1]. This form of ‘othering’, experienced by the patients due to the discriminatory attitudes of some healthcare workers, may not only discourage patients from seeking immediate care, but also displace their trust of the system [36], rendering care which feels to women like a ‘tick box’ exercise and not patient-centred. These blanket assumptions of patients, specifically minority ethnic women in maternity care, can result in dangerous consequences. For example, it has previously been found generalised assumptions such as minority ethnic women having higher pain tolerance or are simply overly demanding [44], are not based in science, but are prejudices which can lens the perception of healthcare professionals, by virtue of the workplace environment and culture they might inhabit.

The underlying, institutional racism in the form of implicit bias, being silenced, and disbelieving patients are examples which set apart this analysis from the one undertaken with maternity healthcare professionals [1], insofar as they are formed on the basis of more overt racism and an expectation by healthcare professionals that minority ethnic women simply cannot or will not perform the role of the ‘perfect patient’ in maternity care [45]. What this does demonstrate, however, is that the cracks in the system are visible not only on the surface (i.e. to the healthcare professionals themselves), but also from within (i.e. by the patients who seek care from the NHS).

In response to the anticipation of this discrimination, seeking out someone who would advocate for them was a tactic employed by many women, who often felt ashamed, not taken seriously, over-looked, and in the case of those who experienced mental health issues, unable to disclose their difficulties due to fear of being dismissed and undermined. Under-represented women’s perinatal mental health has been at the centre of much previous research [11, 42, 46, 47], with fear of stigma often arising as the predominant reason for non-disclosure. Similar to previous work [48–50], findings from the present study suggest that essential steps to care which is being personalised to women’s needs may be perinatal mental health staff within maternity services, as well as positive relationships initiated by midwives and other healthcare professionals.

Participants did not need to have experienced discrimination to anticipate it when coming into contact with maternal health services, which reflects previous research stating that anticipated inequality of treatment can lead to distrust in the service [11, 37, 42, 48, 50]. As found with healthcare professionals in the ‘Imperfect Mosaic’ theory, lack of authenticity and humility resulted in a disconnect between cultures and religions; and women often highlighted an increased likelihood of disclosing and trusting healthcare staff from minority ethnic backgrounds. This lack of diversity and representation amongst healthcare professionals inherently affects how the patient perceives the healthcare worker and it is therefore pivotal for maternal healthcare professionals to be culturally aware and compensate for the lack of diversity in maternal healthcare.

### Implications for practice

**Fig. 1:**
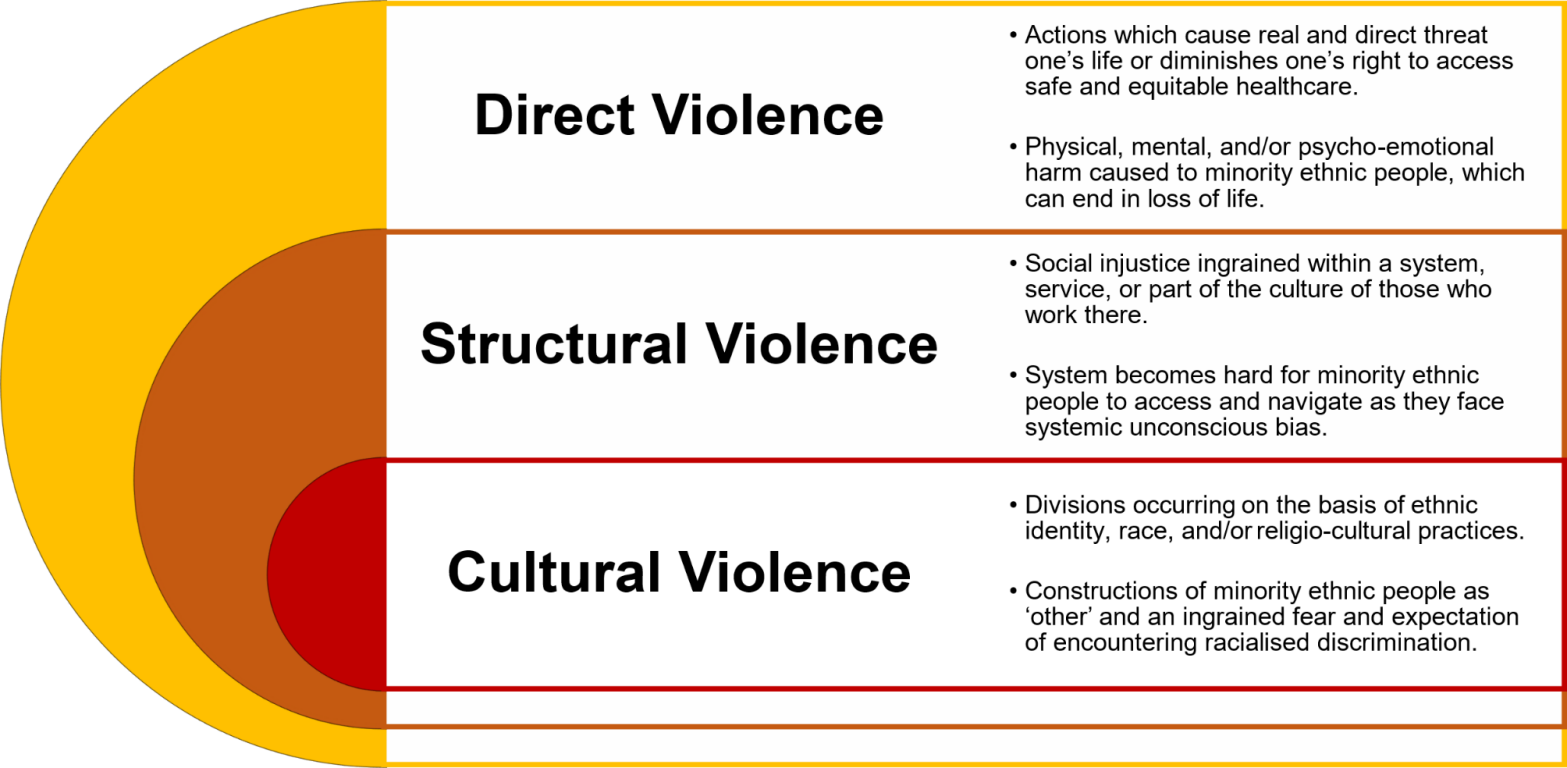
Stacked Venn Diagram explaining degrees of violence against minority ethnic people *N/B.* Diagram draws influence from talk by Kilby & Staniforth [51]

As with healthcare professionals working in NHS maternity services [1], the issues of race and ethnicity from a patient perspective are complex, with no simple solution in evidence. We realise that in drawing attention to issues which have been extant in the literature for generations, there is the potential for ‘stereotype threat’, whereby our empirical work can flood the zeitgeist providing further cognitive burden, and risking confirmation of negative stereotypes with regards to ethnicity and race. However, we would resist this possibility, by instead extending the argument and drawing attention to the layers of violence minority ethnic women may face in UK-based maternity care. We argue that the cultural violence we saw enacted in our previous theory developed with healthcare professionals [1], can become insidious, in turn leading to structural violence, which itself may mediate between cultural and direct or actual violence against minority ethnic groups (see Fig. 1). If these levels of violence go unchallenged, violence will not only continue, but will be perpetuated with both the system and the actors within it becoming implicit in its manifestation and consequences. However, we recognise the importance of providing evidence-based solutions, and so have highlighted from our data solutions which raise the real possibilities for positive change.

First, in taking our findings from the current analysis in relation to those which were previously reported using data from UK maternity and children’s healthcare professional staff [1], we must address unconscious bias amongst healthcare professional staff, who may be influenced and indoctrinated not by personally-held belief systems, but by their workplace culture and environment [49]. It is clear from our results from this work, that women from minority ethnic groups may be more aware of racial disparities within maternity settings [4–7], and may now be approaching maternity care with the expectation of some form of racialised discrimination even if not seen as intentional. Therefore highlighting how unconscious biases may pervade is deemed essential for positive transformation in maternity care settings.

Second, we must recalibrate the balance of power between professional and patient. While the agency of women and bodily autonomy is a principle which applies to all women, particular attention must be paid to this principle when caring for with those from minority ethnic groups. Pregnant women and birthing people are inherently not sick, nor are they inherently vulnerable. Their queries must be addressed throughout antenatal and postnatal care by provision of high-quality, accessible, and culturally sensitive information on how to navigate the complex maternity system, to enable empowerment, informed decision-making, and avoidance of poor labour and birthing experiences [50].

Third, we must reinforce notions of equity (i.e., a move towards personalised, relational care; understanding that some people will require different approaches to care delivery to make them feel physically and psychologically safe) over equality (i.e., providing the same care for everyone as a proxy for non-discriminatory care provision) within maternal healthcare. Whilst avoiding stereotypes, such as minority ethnic group women as ‘strong’ or ‘difficult’, healthcare professionals must support and understand the diverse needs of women from minority ethnic backgrounds, which differ from those of the majority ‘White British’ women. Staff must be sensitive to cultural perceptions of pregnancy, birth, and perinatal mental health and wellbeing, whilst engendering a climate of genuine cultural safety (i.e. the ability to access safe healthcare, regardless of one’s ethnic, racial, or religious identity and without fear of worse treatments or prognoses based on these characteristics) [52].

### Strengths, limitations, & future research

Strengths of our study include the design, aimed to collect experiential data from a diverse population of women across the UK, particularly with regards to ethnicity. We had considerable feedback throughout the research from Patient and Public Involvement and Engagement [PPIE]. Our data-driven analysis using grounded theory, rather than a predetermined deductive analysis is also a strength. Our interviews were carried out by various team members, including a peer researcher who had lived experience similar to other participants, which allowed for real-time ‘member-checking’ throughout the design, data collection, analysis, and write-up. Without the peer researcher, we would have had to accept that none of the authors would have had recent maternity care experiences as a limitation of the study. The fact that the peer researcher was an integral part of the team from study design to dissemination, enabled us to sense-check and be declarative in our conclusions made.

Limitations of our study include, as with much qualitative research, concerns about the generalisability of our findings, as we may not have reached particularly socially-deprived women, those suffering from digital poverty, or those with high levels of social complexity. We acknowledge that our participants were slightly older than average for childbearing women in the UK (*M*_*Age*_ = 36 years, of the 13 women who reported their age) and are mostly educated to degree level or above (n = 11/14, with three participants not reporting their educational status). This could have affected our findings insofar as the women in our study may have had more maternity experiences and may have been more aware of the climate surrounding ethnicity in maternity care, hence may have anticipated more issues arising or had more concerns than other members of their respective communities who were younger or less well-educated. We cannot know this for sure, however, as participants were not asked about their knowledge of maternity and/or ethnicity reports within the NHS. We do, however, have representation from various places across the country, meaning our results are widely applicable to care across UK maternity services. However, it is important to recognised that younger, less educated women may also experience racially-biased care, and may be less confident, agentic, or able to advocate for themselves [53].

Future studies should pursue similar research aims in different populations (including in countries where the healthcare system differs), or indeed in other contexts (such as other healthcare services outside of maternal healthcare). Given our Grounded Theory Analysis approach, the – now two – extant theories relating to both the healthcare professional and patient perspectives of the ‘Imperfect Mosaic’ are ripe for testing in future research. We also lend support to previous calls who argue for better inclusivity of migrant women, those who speak little-to-no English, and those women for whom English is not their first language [11, 42, 54, 55].

## Conclusion

In summary, disparities in maternity care continue and action is required to address them. Our findings suggest that such actions should address unconscious bias amongst healthcare professional staff, and emphasise mindfulness of the principle of autonomy and agency among minority-ethnic group women. This may require an emphasis on equity rather than equality, to improve outcomes and experiences of minority ethnic women in maternity care.

## Data Availability

The data supporting the findings of this study are available upon reasonable request from the corresponding author. The data are not publicly available due to privacy or ethical restrictions.

## Declarations

### Patient and public involvement and engagement

From conception of the project, throughout design, planning, recruitment, data collection, analysis, and write-up, we have worked alongside national expert and lay stakeholders with lived experience of pregnancy and childbirth, who formed the Community Advisory Group for The Rep All Women Study which met between March 2021 and January 2022. One of these members [ZB] also acted as a peer researcher on the study. This project was adopted by the National Institute for Health and Care Research Applied Research Collaboration in South London’s Maternity and Perinatal Mental Health Research Theme, and was presented at a meeting of their PPIE group in June 2021.

## Abbreviations

Brexit: The Withdrawal of the United Kingdom from the European Union
NHS: National Health Service
PPIE: Patient and Public Involvement and Engagement
SARS-CoV-2: Severe Acute Respiratory Syndrome Coronavirus 2 (a.k.a. COVID–19)
UK: United Kingdom
